# Brief Report: A Cross-Sectional Study of Anxiety Levels and Concerns of Chinese Families of Children With Special Educational Needs and Disabilities Post-first-wave of COVID-19

**DOI:** 10.3389/fpsyt.2021.708465

**Published:** 2021-09-20

**Authors:** Xueyun Su, Ru Ying Cai, Mirko Uljarević, Jo Van Herwegen, Daniel Dukes, Yufang Yang, Xiaomei Peng, Andrea C. Samson

**Affiliations:** ^1^Department of Early Childhood Education, Faculty of Education and Changning Maternity and Infant Health Hospital, East China Normal University, Shanghai, China; ^2^Aspect Research Centre for Autism Practice, Autism Spectrum Australia, Melbourne, VIC, Australia; ^3^School of Psychological Sciences, Monash University, Melbourne, VIC, Australia; ^4^Faculty of Medicine, Melbourne School of Psychological Sciences, Dentistry, and Health Sciences, University of Melbourne, Melbourne, VIC, Australia; ^5^Department of Psychology and Human Development, Institute of Education, University College London, London, United Kingdom; ^6^Institute of Special Education, University of Fribourg, Fribourg, Switzerland; ^7^Swiss Center for Affective Sciences, University of Geneva, Geneva, Switzerland; ^8^Faculty of Psychology, Unidistance Suisse, Brig, Switzerland

**Keywords:** COVID-19, pandemic, China, SEND, children, anxiety

## Abstract

The COVID-19 pandemic has a multifaceted impact on mental health due to ill health, restrictions and lockdowns, and loss of employment and institutional support. COVID-19 may disproportionally impact families with special educational needs and disabilities (SEND) due to the already higher prevalence of mental health conditions in children with SEND and their parents. Therefore, it is essential to determine the short-term impact of the pandemic on the mental health of families with SEND in order to identify their ongoing health support needs. The current study aims to examine the anxiety level and concerns of children with SEND and their parents living in China. The sample consisted of 271 parents of children with SEND aged between 6 and 17 years (*M*_*age*_ = 8.37; *SD*_*age*_ = 2.76). Parents completed an online survey between 10 April to 8 June 2020. Both child and parental anxiety levels and various concerns increased after the initial wave of COVID-19 when compared with retrospective pre-COVID-19 levels. Parental anxiety and concern levels were significantly higher for those living in rural areas compared to urban areas. In addition, parental and child anxiety and concern levels were significantly correlated with each other. Parental anxiety at the lowest level made a unique and significant statistical contribution to children's anxiety levels. The implications of the study findings are discussed.

## Introduction

Historically, epidemics and pandemics such as Ebola and severe acute respiratory syndrome (SARS) have led to increased rates of psychopathology, including anxiety, depression, and post-traumatic symptoms (1, 2). The increased prevalence of psychopathology is also true for the recent SARS-CoV2 (hereafter COVID-19) pandemic (3). A systematic review and meta-analysis found the global prevalence of stress, anxiety, and depression during the first few months of the COVD-19 pandemic were 29.6, 31.9, and 33.7%, respectively (4). These rates are much higher than the prevalence of mental illnesses before the start of the pandemic, even in conflict-affected populations [e.g., (5)].

The reasons why the COVID-19 pandemic negatively impact people's mental health are multifacted. Many people have experienced significant loss during the pandemic (6, 7). As of August 2021, there were more than 200 million cases of COVID-19 and the global death toll has surpassed 4.3 million and continues to rise. Reports suggest that even for people who have recovered from COVID-19, there might subsequently be a higher risk of having chronic cardiovascular damage (8) and psychiatric illness (9). Other non-health-related losses include loss of livelihood and financial well-being due to unemployment, as well as loss of freedom of movement and social interactions due to quarantine and restrictions, which has been shown to impact the mental health of affected individuals negatively (10, 11). Apart from the mental health effects of losses related to COVID-19, misinformation and experiencing fear of the COVID-19 outbreak itself (or coronaphobia) also lead to feelings of helplessness, loneliness, depression, generalized anxiety, and death anxiety (12, 13). Most of these studies were conducted in general populations. The mental health impact on children with special educational needs and disabilities (SEND) and their families are largely overlooked, despite them being among the most vulnerable population.

The COVID-19 pandemic may impact the mental health of families with children with SEND disproportionally due to the already higher prevalence of mental health conditions in children with SEND and their parents (14). In non-COVID-19 circumstances, parents of children with SEND report poorer mental health than those of neurotypical children (15, 16). Factors that lead to poorer parental mental health include elevated stress levels (17, 18), lack of social support (19) and use of less effective coping styles (20). In addition to parents of children with SEND experiencing poorer mental health, children with SEND also often report higher levels of psychopathology in normal circumstances. For example, the prevalence of psychiatric conditions in autistic children is high, with one study finding 70% of participants had at least one comorbid condition (21). Dimensional factors found to impact the mental health of children with SEND include emotion dysregulation (22, 23), lower executive functioning (24), and higher intolerance of uncertainty (25). Furthermore, there is evidence for a bi-directional relationship between parental and child anxiety (26, 27), which may heighten the mental health issues experienced by families with children with SEND. Recent research in the UK found that awareness of COVID-19 and parental anxiety predicted child anxiety levels during the pandemic (28).

Furthermore, the COVID-19 situation can exacerbate the poor mental health experienced by families with children with SEND by disruption of daily routines, the disintegration of support networks including individual therapy, parents juggling both work and homeschooling, and increased behavioral problems in children (14, 29, 30). Indeed, initial evidence suggests families of children with SEND experienced poor mental health during the pandemic. A large proportion of families reported increased anxiety and fear during the pandemic (14); specifically, both parents and children with SEND were experiencing loss, worry, and changes in mood and behavior due to the changes resulting from the pandemic. Caregivers of individuals with an intellectual disability (ID) reported significantly greater levels of anxiety, depression, and feelings of defeat or entrapment when compared with parents of children without disabilities (31). Even when parents reported positive aspects of the pandemic, such as being together as a family, they raised concerns about the long-term impacts of the loss of services, education, social opportunities on their children's development (32). Parental concern about the long-term consequences is likely to increase parental stress in the future as the pandemic continues. In addition to the higher prevalence of mental distress experienced by families of children with SEND, people with SEND, such as people on the autism spectrum, also experienced significant barriers accessing COVID-19 services due to healthcare inequalities (33).

It is crucial to determine the short- to long-term impact of the pandemic on families' mental health to identify the ongoing health support needs to provide adequate support for parents and the person for whom they are the principal caregivers. Until now, most studies that examined the mental health impact of the pandemic on families with children with SEND were conducted in countries where case numbers were increasing, which meant we could understand the immediate mental health effects of COVID-19. Here, we are interested in exploring the short-term impact of the pandemic. China had been able to mostly control the pandemic in 2020, since late April. Therefore, it would be feasible to study the short-term effect of the pandemic on the mental health of families with children with SEND through surveying families living in China about their mental health post-April 2020.

None of the reviewed studies focused on Chinese families with children with SEND and there is a paucity of research on this group. Tamo (34) examined the maternal stress levels during- and pre-COVID-19 pandemic in China and found that mothers reported more stress during the pandemic when using the Parenting Stress Index-Short Form (PSI-SF) (35). Although authors noted that mothers living in rural areas (*M*_*PSI*−*SF*_ = 69.21; *SD* = 2.72) reported less stress than those in urban areas (*M*_*PSI*−*SF*_ = 70.69; SD = 2.25) during the pandemic, this observation was not based on formal statistical analysis. Chen et al. (36) studied the mental health of parents of children with autism, intellectual disability and visual or hearing impairment during the pandemic. They found that parents of children with autism were more likely to have mental health problems than parents of children with visual or hearing impairment or intellectual disability.

The current study aims to examine the anxiety levels and concerns of Chinese parents and their children with SEND after an initial wave of the pandemic. It is hypothesized that parental and child anxiety levels would be higher after the initial wave of COVID-19 (hereby post-COVID-19) compared to the levels prior to COVID-19 (pre-COVID-19) using retrospective reports. We also explored a range of health- and life-related concerns of parents and children and examined whether the anxiety and concern levels of parents post-COVID-19 were influenced by residence locality (rural or urban) or disability types. Additionally, we envisaged that parent and child anxiety and concern levels would be significantly associated with each other. Finally, we explored whether child demographics, parental anxiety, and child verbal ability and awareness of COVID-19 predicted child anxiety levels.

## Methods

### Participants

Participants were 271 Chinese parents (*M*_*age*_ = 36.05; *SD*_*age*_ = 5.54; range = 18–50 years) of children with SEND aged 6 to 17 years (*M*_*age*_ = 8.37; *SD*_*age*_ = 2.76; 30% female; see Table 1 for demographic information). The three most frequently reported diagnoses of the children with SEND were autism spectrum disorder (ASD; 38%), intellectual disability (27%), and language and speech disorder (13%). Parents were mostly mothers (81%). Almost two-thirds of the individuals with SEND (66%) lived in urban areas, while the rest lived in rural areas. Over half of the children were non-verbal (55%), and more than a third were aware of COVID-19 (41%). The inclusion criteria was that parents needed to have a child with SEND. Although data was collected from parents of children of all ages, in this analysis, we have focused on parents of children aged between 6 and 17 to maintain homogeneity in our sample, as concerns of parents with very young or adult children might substantially differ from concerns of parents with school-aged children.

**Table 1:** Demographic information of parents and children with SEND.

**Parents**	***n* (%)**	**Children with SEND**	***n* (%)**
**Gender**		**Gender**	
Female	217 (80%)	Female	82 (30%)
Male	51 (19%)	Male	189 (70%)
Unspecified	3 (1%)		
**Parent type**		**Residence**	
Mother	220 (81%)	Urban	179 (66%)
Father	51 (19%)	Rural	92 (34%)
**Highest level of education**		**Diagnoses**	
No formal education	40 (15%)	Autism spectrum disorder	102 (38%)
Middle school	3 (1%)	Intellectual disability	74 (27%)
High school	57 (21%)	Language and speech disorder	35 (13%)
Vocational and technical school	89 (33%)	Down syndrome	19 (7%)
Bachelor degree	67 (25%)	Developmental delay	10 (4%)
Postgraduate degree	12 (4%)	Dyspraxia/Developmental coordination disorder	11 (4%)
Missing	3 (1%)	Attention deficit or attention deficit hyperactivity disorders	8 (3%)
		Cerebral palsy	3 (1%)
		Hearing impairment	3 (1%)
		Other syndrome or diagnosis	6 (2%)
**Employment status**		**Living arrangement**	
Full-time employment	90 (33%)	At home with family	258 (95%)
Part-time employment	17 (6%)	In a group home	2 (1%)
Homemaker	141 (52%)	In a supported living setting	4 (2%)
Unemployed	13 (5%)	On his/her own	2 (1%)
Student	1 (0%)	With a significant other	1 (0%)
Other	5 (2%)	Missing	4 (1%)
Missing	4 (2%)		

*SEND, Special Educational Needs and Disabilities*.

### Procedures and Measures

Ethics approval was obtained from UniDistance Suisse, Switzerland, for the larger study titled, *How Families with Children with Special Needs are coping with the COVID-19 Pandemic: An International Online Study*. This international study was initially designed by Van Herwegenet al. (37) (Principal Investigators) in English, German and French. The study involved participants completing an online survey (Qualtrics). The Principal Investigators invited internationally researchers from 25 countries to collaborate for the study, which resulted in the translation into 16 different languages, including Arabic and Spanish. For the Chinese survey, the English version was translated into Chinese by the first author, a professor with a doctoral degree in Special Education and has worked in an English-speaking country. The Chinese version of the survey was then back-translated by a master student in Special Education who is studying in the USA to ensure the consistency of Chinese and English versions. Five parents of children with SEND were recruited to complete the Chinese survey and gave feedback to make the language and terminology easy to understand. A convenience sampling method was used to recruit Chinese parents of children with SEND to participate in this study. The first author contacted principals and teachers to recruit parents and caregivers from public special schools, private early intervention centers, and rehabilitation centers of local China Disabled People's Federation in various places such as Shanghai, Shandong Province, Henan Province, Zhejiang Province, Anhui Province, and Guangzhou Province. Principals and teachers were contacted *via* phone or WeChat. The first author also posted messages to parent support groups *via* WeChat. Interested individuals were sent the survey link. Data collection for China occurred between 10 April and 8 June 2020. Once participants clicked on the survey link, instructions for completing the questionnaire were displayed.

The survey consisted of 111 open- and closed-ended questions and extra questions that were displayed only in dependence of a specific response.

We describe here the questions that are relevant for the current study only. Participants were asked questions about their SEND child's background, including demographic information (e.g., gender, age, parent's level of education and employment status, urban, or rural residence), verbal ability, and the SEND child's medical background (e.g., clinical diagnosis, related health issues). The main part of the survey focused on anxieties and specific concerns of the participating parent as well as anxieties and concerns of their child with SEND. Parents were asked to identify on a scale from 1 to 5 (with 1 = *not anxious at all* to 5 = *very anxious*) their anxiety level and the level of anxiety that their child with SEND experienced. To measure concerns, parents were asked to identify on a scale from 1 to 5 (with 1 *not worried at all* to 5 being *very worried*) their concerns and the level of concerns that their child with SEND experienced. The questions around concerns were linked to a range of health- and life-related domains such as concerns about COVID-19, their child's safety concerning COVID-19 (for child: questions refers to family's safety), lower levels of social contact of their child, their child not being able to approach others, their child's ability to cope with changes in routine, their child becoming bored, the possibility of parent getting ill (for child, question refers to others getting ill), the loss of institutional support for child, and the potential for family conflict. For each anxiety and specific concern question, the survey asked respondents to provide an answer related to three different time points: (1) before the pandemic began (pre-COVID-19), (2) when COVID-19 first started in their country, and (3) at the time the participant completed the survey (post-COVID-19), which varied in time. For the purpose of the present study, we focused on two time points: pre- and post-COVID-19. The survey also asked parents whether their children were aware of COVID-19. Some of these questions asked about two time points (before COVID-19 and now) and some about 3 time points (before COVID-19, at the beginning of COVID-19, now). An additional attention check question was included in the survey to ensure participant responses were valid. Participants who failed the attention check were removed. The questionnaire took ~30 min to complete. At the end of the survey, parents were asked to input an identification code (initials of their name and date of birth). This code allowed participants to withdraw their anonymous data at any point after completing the survey if they wished to do so. The identification codes were checked to ensure no parent completed the survey more than once.

### Analysis Plan

Data was first screened to ensure there were no invalid responses (e.g., age had to be a valid number) and participants missing age (child or parent), gender (child or parent), parent type or residence information were removed. After the cleaning procedure, data from 271 participants were used for the analysis. Wilcoxon Signed Ranks Tests were conducted to examine the changes in anxiety and concern levels of parents and children pre- and post-COVID-19. Mann–Whitney *U*-tests for independent samples and Kruskal-Wallis tests were used to assess the differences in parental anxiety and concerns levels based on residence (rural or urban) and child disability type. Finally, a Speakman's Rank-Order correlation analysis was used to examine the associations between parent and child anxiety and concern levels. Ordinal regression was used to determine whether child demographics (age, gender, and residence), child verbal ability, child awareness COVID-19, and parental anxiety level predicted variance in child anxiety level. A *p* < 0.05 indicated statistical significance. Missing data was addressed by excluding cases either pair-wise (correlations), list-wise (Ordinal regression), or test-by-test (Wilcoxon Signed Rank Test). SPSS Statistics version 21 for Mac was used for all statistical analyses.

## Results

### Anxiety and Concern Levels Before and After an Initial Wave of COVID-19

The changes in anxiety and concern levels of parents and children as reported by parents are listed in Table 2.

**Table 2:** Anxiety and concern levels: before and after initial wave of COVID-19 and correlations between parent and child.

	**Parent**				**Child**				**Correlations** **between child and** **parent**		
	***n***	***Z***	***p***	**Effect size**	***n***	***Z***	***p***	**Effect size**	***n***	***r***	***p***
Anxiety level	211	−3.11	0.002	0.214	201	−2.83	0.005	0.200	194	0.385	0.000
Concern about COVID-19	215	−1.65	0.099	–	199	−3.77	0.000	0.267	197	0.463	0.000
Concern about child's safety with respect to COVID-19 (for child, question refers to family's safety)	210	−1.38	0.167	–	202	−2.22	0.026	0.156	197	0.401	0.000
Concern about child's health	212	−0.739	0.460	–	200	−2.66	0.008	0.189	196	0.329	0.000
Concern about lower levels of social contact of child	209	−3.08	0.002	0.213	200	−3.32	0.001	0.235	198	0.391	0.000
Concern about child not being able to approach others	209	−2.08	0.038	0.144	203	−3.37	0.001	0.236	194	0.341	0.000
Concern about child's ability to cope with changes in routine	206	−1.45	0.147	–	200	−3.03	0.002	0.214	190	0.301	0.000
Concerns about child becoming bored	212	−2.11	0.035	0.145	199	−3.83	0.000	0.271	192	0.368	0.000
Concerns about the possibility of parent getting ill (for child, question refers to others getting ill)	209	−2.43	0.015	0.168	198	−2.10	0.036	0.149	191	0.292	0.000
Concerns about loss of institutional support for child	208	−5.14	0.000	0.356	199	−3.68	0.000	0.261	194	0.397	0.000
Concerns about family conflict	207	−0.55	0.581	–	200	−1.39	0.164	–	195	0.392	0.000
Concerns about their financial situation	207	−4.98	0.000	0.346	196	−0.83	0.407	–	190	0.352	0.000

Both child and parental anxiety levels increased post-COVID-19, with small effect sizes. Parents' concerns about the possibility of themselves getting ill and the concerns around social relationships, boredom, and institutional support of their children increased significantly. Parents' concern about their financial situation also increased with medium effect. Parents reported that their child's concerns on most aspects of life significantly increased with small effect, except child's concern on family conflict and their financial situations, which showed no significant effect.

### Associations of Child Residence and Child Disability Types With Parental Anxiety and Concern Levels

Parental anxiety and concern levels were significantly higher for those living in rural areas compared to urban areas: anxiety (*U* = 4,071, *z* = −2.277, *p* = 0.023, and *r* = 0.157), COVID-19 (*U* = 4,320, *z* = −2.156, *p* = 0.031, and *r* = 0.147), child's safety with respect to COVID-19 (*U* = 4,199, *z* = −2.000, *p* = 0.045, *r* = 0.137), child's ability to cope with changes in routine (*U* = 3,917, *z* = −1.975, *p* = 0.048, *r* = 0.138), child's boredom (*U* = 3,977, *z* = −2.580, *p* = 0.010, and *r* = 0.177), and family conflict (*U* = 4,111, *z* = −1.982, *p* = 0.047, and *r* =0.137). A few concerns were close to significance: child's health (*U* = 4,258, *z* = −1.901, and *p* = 0.057), child's reduced social contact (*U* = 4,204, *z* = −1.878, and *p* = 0.060), possibility of self getting ill (*U* = 4,109, *z* = −1.888, *p* = 0.059), and own financial situation (*U* = 4,038, *z* = −1.897, and *p* = 0.058).

Parental anxiety and concern levels across the child disability types were also examined and no significant differences were found.

### Correlations of Anxiety and Concern Levels and Predictors of Child Anxiety

Parent and child anxiety and concern levels were all significantly associated with each other, with medium to large effects (see Table 2).

An ordinal regression model with child anxiety level as the dependent variable and child verbal ability, child awareness of COVID-19, and parental anxiety level as independent variables was statistically significant [$\chi_{\left(9\right)}^{2}$ = 25.71, *p* = 0.002]. Both Pearson's [$\chi_{\left(551\right)}^{2}$ = 538.908, *p* = 0.636] and Deviance [$\chi_{\left(551\right)}^{2}$ = 310.459, *p* = 1.000] chi-square tests indicated the model fits the data well. Test of parallel lines showed that the parameters are the same for all categories held [$\chi_{\left(27\right)}^{2}$ = 23.983, *p* = 0.631]. The model as a whole explained between 11 and 13% (Cox and Snell, and Nagelkerke R square) of the variance in child anxiety. As shown in Table 3, only parental anxiety at the lowest level made a unique statistically significant contribution to the model (β = −1.828, *p* = 0.015).

**Table 3:** Ordinal regression predicting child anxiety level.

	***B***	**SE**.	**Wald**	**df**	***p***	**95.0% CI for odds ratio**	
						**Lower**	**Upper**
Child age	0.010	0.060	0.029	1	0.864	−0.107	0.128
**Gender**							
Male	0.430	0.354	1.480	1	0.224	−0.263	1.123
**Residence**							
Urban	0.225	0.342	0.435	1	0.509	−0.444	0.895
**Child verbal ability**							
Non-verbal	−0.045	0.326	0.019	1	0.891	−0.684	0.594
**Child COVID-19 awareness**							
Not aware	−0.261	0.338	0.593	1	0.441	−0.924	0.403
**Parental anxiety level**							
1 (Not at all)	−2.187	0.800	7.478	1	0.006^*^	−3.754	−0.620
2	−0.880	0.798	1.217	1	0.270	−2.444	0.684
3	−0.875	0.807	1.175	1	0.278	−2.456	0.707
4	0.609	0.884	0.475	1	0.491	−1.123	2.341

* *p < 0.01*.

## Discussion

This study aimed to examine the anxiety levels and concerns of Chinese parents and their children with SEND after the initial wave of the pandemic that occurred between December 2019 and April 2020. As hypothesized, using retrospective reports, parental and child anxiety levels post-COVID-19 were significantly higher when compared to the levels pre-COVID-19. This finding is in line with the existing research findings that indicate parents and children with SEND report increased anxiety and fear during the pandemic (14). Further, caregivers of children with SEND reported greater levels of mental illnesses when compared with parents of children without disabilities (31). Thus, our finding suggest the poor psychological wellbeing of parents and their children with SEND are unlikely to improve after the initial wave of the pandemic.

In addition to examining the parental and child anxiety levels, we explored a range of health- and life-related concerns of parents and children. The types of concerns that increased post-COVID-19 for parents were slightly different from those reported for their children. Parental concerns were mainly around the pandemic's impact on their children's daily life, specifically reduced social interactions, increased boredom, and loss of institutional support. Although the effects of reduced social interactions due to the pandemic on children's growth and development are still unknown, parental concerns about their children's reduced social interactions are not unfounded. Critical periods in child development are essential for developing social skills and theory of mind (38). China's Ministry of Education closed all schools during the winter break in January 2020, just before the 2020 spring semester began, impacting 278 million students across primary and postsecondary schools (39). Students returning to school occurred incrementally between April and June 2020, depending on the year level of students. School closure would have also impacted children with SEND attending special schools. Parents' concerns identified in our study were similar to concerns of parents of children with SEND living in other countries such as the USA and UK, which were concerns about the long-term impacts of the loss of services, education, social opportunities on their children's development (28, 32). Chinese parents were also concerned about their financial situation. Over the last 20 years, China's unemployment rate peaked in early 2020 (40). Additionally, the pandemic and associated restrictions particularly impact migrant workers; up to 50 million migrants lost their jobs in late March 2020 (41). Although parents were concerned about the possibility of getting ill themselves, they were not concerned about the pandemic's impact on children's health, possibility due to children having less severe symptoms when infected (42).

Parents reported that children's concerns about most aspects of life significantly increased after the initial wave of COVID-19. Like parents, children were more concerned about their reduced social interactions, increased boredom, and loss of institutional support. Children were also more concerned about the possibility of others getting ill. Interestingly, children's concern about their own health increased, which may be explained by their fears and worries about catching the virus because they think they can infect their grandparents (43). Children were not concerned about family conflict or their financial situation. Since the children were all under 18 years, it is unlikely for them to be financially responsible. Given the rise in domestic violence cases in parts of China (44), it was interesting to observe that parents did not report increased concerns on family conflict for themselves or for their children.

We also examined whether the anxiety and concern levels of parents post-COVID-19 were associated with residence locality (rural or urban) and disability types. Parents living in rural areas were significantly more anxious and more concerned about COVID-19, their child's safety with respect to COVID-19, child's ability to cope with changes in routine, and family conflict. These results did not align with Tamo's (34) study, which showed no differences in maternal stress levels between mothers living in rural and urban areas. This discrepancy may be due to the use of different measures: we used measures of anxiety and concerns, while Tamo used a measure of stress. These elevated anxiety and concern levels of rural parents are likely to result from rural residents performing fewer preventive behaviors against COVID-19 and low levels of local government aid and financial subsidies offered (45, 46). The fact that the rural residents who are primarily mothers were more concerned about family conflict might be explained by pre-COVID-19 research indicating the rate of intimate partner violence (male-to-female) is higher in rural settings (47). There is also a strong tradition of patriarchy in rural China (48). We did not find any significant differences on parental anxiety and concerns across the various disability types.

We found that the anxiety and all concerns of parents and children with SEND were significantly correlated. In addition, parental anxiety, especially at the lowest level, statistically predicted variance in child anxiety level, which suggests children of parents that were not at all anxious had significantly lower anxiety. Our findings align with previous research that showed bi-directional relationships between parental and child anxiety during pre-COVID-19 settings (26, 27) and parental anxiety predicting the anxiety of children with SEND during COVID-19 (28). Other child characteristics such as age, gender, verbal ability, and awareness of COVID-19 did not predict child anxiety levels. The observation on awareness of COVID-19 differed with the findings from the same international study using a sample of parents living in the UK. Sideropoulos et al. (28) found that awareness of COVID-19 significantly predicted child anxiety. It is worth noting that the age range of the children in the UK sample ranged between 1 and 45 years, which may have contributed to the different observations across the two samples.

There are several limitations of the study that require mentioning. First, the study relied on parent reports of children's diagnoses; therefore, it was not possible to confirm the clinical diagnoses of the children with SEND. However, parents were recruited through channels where children must have special needs and disabilities (diagnosed by qualified physicians from public hospitals), including special schools and private early intervention centers. Therefore, all the children included in this study had general SEND, and the overall observations hold, even if the conclusions based on transdiagnostic comparisons might need to be treated with more caution. Second, although the study's cross-sectional design allowed us to explore the relationships between anxiety and concern levels of parents with child residence and disability types, it does not allow us to infer causal relationships. Other limitations of the study include the use of convenience sampling rather than random sampling, having a relatively small sample size, and non-rigorous pre-design.

Despite these limitations, this study has several important implications. The study provides evidence that not only did the anxiety and concern levels of children with SEND and their parents increased after the COVID-19 pandemic through retrospective reports, but importantly, the findings also suggest that these anxiety and concern levels are unlikely to reduce after the initial wave of the pandemic. For places with more than one epidemic waves, research has shown that anxiety levels of people tend to have a decreasing trend [e.g., (49)]. Our study has identified key sources of stress that impact the mental health of Chinese parents and their children. We have also shown the influence of parental anxiety on child anxiety. Some of our study findings such as parental concerns around children's reduced social interactions are likely to be experienced by parents and children living in other countries or cities, since school closures occurred across the globe. While other findings such as increased anxiety and concern levels experienced by rural families may be China specific, due to great rural-urban disparities. Further research is needed in order to fully characterize anxiety trends and predictors across different contexts, countries and waves. Nevertheless, our study highlights the urgent need to provide support for Chinese children with SEND and their parents and important information in terms of specific areas where support is particularly needed. Any mental health interventions for reducing the anxiety of children with SEND should also include improving parents' mental health.

## Data Availability Statement

The original contributions presented in the study are included in the article/supplementary material, further inquiries can be directed to the corresponding author/s.

## Ethics Statement

The studies involving human participants were reviewed and approved by UniDistance Suisse. Written informed consent for participation was not required for this study in accordance with the national legislation and the institutional requirements.

## Author Contributions

JV, DD, and AS designed the study with input from MU and RC. XS collected the data. XS, RC, YY, and XP had access to the data. RC, YY, and XP conducted the analyses. RC drafted the initial manuscript. All authors critically reviewed, provided feedback on the initial version of the manuscript, and approved the final version of the manuscript.

## Funding

Swiss National Science Foundation (PP00P1_176722 for Andrea Samson), Research Funds of the Unidistance Suisse. XS is funded by Changning Maternity and Infant Health Hospital, East China Normal University for project titled Research on Development and Assessment of Children in Early Childhood. MU is currently supported by the Australian Research Council Discovery Early Career Researcher Award (DE180100632). AS has the Swiss National Science Foundation professorship (PP00P1_176722).

## Conflict of Interest

The authors declare that the research was conducted in the absence of any commercial or financial relationships that could be construed as a potential conflict of interest.

## Publisher's Note

All claims expressed in this article are solely those of the authors and do not necessarily represent those of their affiliated organizations, or those of the publisher, the editors and the reviewers. Any product that may be evaluated in this article, or claim that may be made by its manufacturer, is not guaranteed or endorsed by the publisher.
